# Tetra­kis(μ_2_-2-phen­oxy­propionato)-κ^3^ *O*,*O*′:*O*′;κ^3^ *O*:*O*,*O*′;κ^4^ *O*:*O*′-bis­[(1,10-phenanthroline-κ^2^ *N*,*N*′)(2-phen­oxy­propionato-κ^2^ *O*,*O*′)gadolinium(III)]

**DOI:** 10.1107/S1600536811036130

**Published:** 2011-09-14

**Authors:** Jin-Bei Shen, Jia-Lu Liu, Guo-Liang Zhao

**Affiliations:** a College of Chemistry and Life Science, Zhejiang Normal University, Jinhua 321004, Zhejiang, People’s Republic of China; b Zhejiang Normal University Xingzhi College, Jinhua, Zhejiang 321004, People’s Republic of China

## Abstract

In the centrosymmetric binuclear title complex, [Gd_2_(C_9_H_9_O_3_)_6_(C_12_H_8_N_2_)_2_], the two Gd(III) ions are linked by four 2-phen­oxy­propionate (*L*) groups in bi- and tridentate bridging modes. Each Gd^III^ ion is nine-coordinated by one 1,10-phenanthroline mol­ecule, one bidentate chelating carboxyl­ate group and four bridging carboxyl­ate groups in a distorted GdN_2_O_7_ monocapped square-anti­prismatic geometry.

## Related literature

For background to phen­oxy­alkanoic acids, see: Markus & Buser (1997 ▶). For a related Gd complex, see: Yu *et al.* (2010 ▶). For isotypic structures, see: Shen *et al.* (2011*a* ▶) for Tb; Shen *et al.* (2011*b* ▶) for Pr; Shen *et al.* (2011*c* ▶) for Dy; Shen *et al.* (2011*d* ▶) for Ce; Shen *et al.* (2011*e* ▶) for Ho; Shen *et al.* (2011*f* ▶) for La.
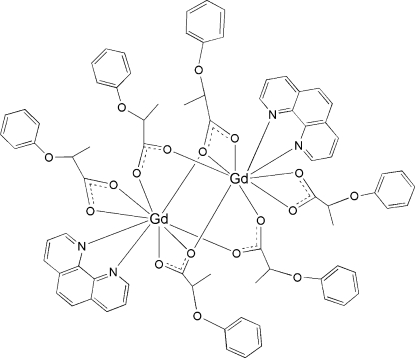


## Experimental

### 

#### Crystal data

[Gd_2_(C_9_H_9_O_3_)_6_(C_12_H_8_N_2_)_2_]
*M* *_r_* = 1665.88
Monoclinic, 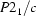

*a* = 11.4876 (2) Å
*b* = 25.8305 (4) Å
*c* = 13.8715 (2) Å
β = 120.554 (1)°
*V* = 3544.58 (10) Å^3^
*Z* = 2
Mo *K*α radiation
μ = 1.93 mm^−1^
*T* = 296 K
0.49 × 0.24 × 0.16 mm

#### Data collection

Bruker APEXII CCD diffractometer
Absorption correction: multi-scan (*SADABS*; Sheldrick, 1996 ▶) *T*_min_ = 0.585, *T*_max_ = 0.741
47235 measured reflections
6250 independent reflections
5788 reflections with *I* > 2σ(*I*)
*R*_int_ = 0.022

#### Refinement

*R*[*F* ^2^ > 2σ(*F* ^2^)] = 0.021
*wR*(*F* ^2^) = 0.043
*S* = 1.15
6250 reflections
463 parameters
H-atom parameters constrained
Δρ_max_ = 0.72 e Å^−3^
Δρ_min_ = −0.37 e Å^−3^

### 

Data collection: *APEX2* (Bruker, 2006 ▶); cell refinement: *SAINT* (Bruker, 2006 ▶); data reduction: *SAINT*; program(s) used to solve structure: *SHELXS97* (Sheldrick, 2008 ▶); program(s) used to refine structure: *SHELXL97* (Sheldrick, 2008 ▶); molecular graphics: *XP* in *SHELXTL* (Sheldrick, 2008 ▶) and *DIAMOND* (Brandenburg, 2006 ▶); software used to prepare material for publication: *SHELXL97*.

## Supplementary Material

Crystal structure: contains datablock(s) I. DOI: 10.1107/S1600536811036130/wm2524sup1.cif

Structure factors: contains datablock(s) I. DOI: 10.1107/S1600536811036130/wm2524Isup2.hkl

Additional supplementary materials:  crystallographic information; 3D view; checkCIF report

## Figures and Tables

**Table 1:** Selected bond lengths (Å)

Gd1—O7^i^	2.3528 (14)
Gd1—O4	2.3624 (15)
Gd1—O5^i^	2.3991 (15)
Gd1—O2	2.4443 (16)
Gd1—O8	2.4742 (15)
Gd1—O1	2.4826 (15)
Gd1—N2	2.5496 (18)
Gd1—N1	2.6184 (18)
Gd1—O7	2.6270 (15)

Symmetry code: (i) 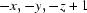.

**Fig. 1.**
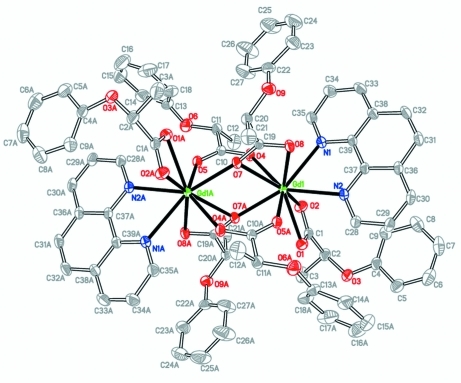
The molecular structure of the title complex, showing the atom-labelling scheme. Displacement ellipsoids are drawn at the 30% probability level.

**Fig. 2.**
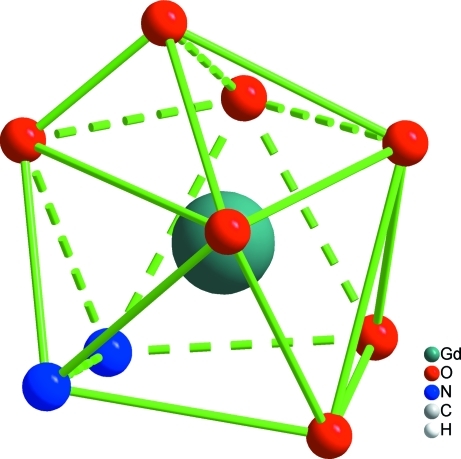
The coordination environment of the Gd(III).

## supplementary crystallographic information

### Comment

The group of phenoxyalkanoic acids includes a considerable number of important herbicides. The desired biological activity is largely dependent on the length of the carbon chain of the alkanoic acid, the nature of the phenoxy group, and the position of its attachment to the carbon chain (Markus & Buser, 1997). The structures of 2-phenoxypropionic acid (H*L*) complexes coupled with their special functionality catched our interest. Here, we describe the Gd^III^ title complex, (I).

The structure of complex (I) is shown in Fig. 1 and the coordination environment of Gd(III) is shown in Fig. 2. The dimeric title compound (I) is centrosymmetric and is comprised of six *L* anions and two phenanthroline ligands. The two Gd(III) ions are linked together by four *L* groups through their bi- and tri-dentate bridging modes, forming a dimeric unit. The distance between two Gd(III) ions in the dimeric unit is 4.0047 (2) Å, which is similar to a related complex (Yu *et al.*, 2010). Each Gd(III) ion is coordinated by nine atoms, five of which are oxygen atoms from the bridging carboxylates, two oxygen atoms from the bidentate chelating carboxylate group, and two nitrogen atoms from a 1,10-phenanthroline molecule. The Gd(III) ion adopts a distorted monocapped square antiprisatic geometry (Fig. 2). The Gd—O distances are all within the range 2.3528 (14)–2.6270 (15) Å, and the Gd—N distances range from 2.5496 (18)–2.6184 (18) Å (Table 1).

For isotypic structures, see: for Tb (Shen *et al.*, 2011*a*), for Pr (Shen *et al.*, 2011*b*), for Dy (Shen *et al.*, 2011*c*), for Ce (Shen *et al.*, 2011*d*), for Ho (Shen *et al.*, 2011*e*), for La (Shen *et al.*, 2011*f*).

### Experimental

Reagents and solvents used were of commercially available quality and without purified before use. 2-Phenoxypropionic acid (1.5 mmol), Gd(NO_3_)_3_^.^6H_2_O (0.5 mmol) and 1,10-phenanthroline (0.5 mmol) were dissolved in 20 ml enthanol, then 10 ml water were added to the above solution. The mixed solution was stirred for 12 h at room temperature. Finally, the deposit was filtered off and the colourless solution was kept in the open air. Colourless crystals were obtained after several days.

### Refinement

The H atoms bonded to C and N atoms were positioned geometrically and refined using a riding model [aliphatic C—H = 0.96 Å (*U*_iso_(H) = 1.5*U*_eq_(C)), aromatic C—H = 0.93 Å (*U*_iso_(H) = 1.2*U*_eq_(C))].

### Crystal data

**Table:**

[Gd_2_(C_9_H_9_O_3_)_6_(C_12_H_8_N_2_)_2_]	*F*(000) = 1676
*M**_r_* = 1665.88	*D*_x_ = 1.561 Mg m^−^^3^
Monoclinic, *P*2_1_/*c*	Mo *K*α radiation, λ = 0.71073 Å
Hall symbol: -P 2ybc	Cell parameters from 9931 reflections
*a* = 11.4876 (2) Å	θ = 1.6–25.0°
*b* = 25.8305 (4) Å	µ = 1.93 mm^−^^1^
*c* = 13.8715 (2) Å	*T* = 296 K
β = 120.554 (1)°	Block, colourless
*V* = 3544.58 (10) Å^3^	0.49 × 0.24 × 0.16 mm
*Z* = 2	

### Data collection

**Table:**

Bruker APEXII CCD diffractometer	6250 independent reflections
Radiation source: fine-focus sealed tube	5788 reflections with *I* > 2σ(*I*)
graphite	*R*_int_ = 0.022
phi and ω scans	θ_max_ = 25.0°, θ_min_ = 1.6°
Absorption correction: multi-scan (*SADABS*; Sheldrick, 1996)	*h* = −13→13
*T*_min_ = 0.585, *T*_max_ = 0.741	*k* = −30→30
47235 measured reflections	*l* = −16→16

### Refinement

**Table:**

Refinement on *F*^2^	Primary atom site location: structure-invariant direct methods
Least-squares matrix: full	Secondary atom site location: difference Fourier map
*R*[*F*^2^ > 2σ(*F*^2^)] = 0.021	Hydrogen site location: inferred from neighbouring sites
*wR*(*F*^2^) = 0.043	H-atom parameters constrained
*S* = 1.15	*w* = 1/[σ^2^(*F*_o_^2^) + (0.0115*P*)^2^ + 2.8127*P*] where *P* = (*F*_o_^2^ + 2*F*_c_^2^)/3
6250 reflections	(Δ/σ)_max_ = 0.002
463 parameters	Δρ_max_ = 0.72 e Å^−^^3^
0 restraints	Δρ_min_ = −0.37 e Å^−^^3^

### Special details

**Table:**

Geometry. All e.s.d.'s (except the e.s.d. in the dihedral angle between two l.s. planes) are estimated using the full covariance matrix. The cell e.s.d.'s are taken into account individually in the estimation of e.s.d.'s in distances, angles and torsion angles; correlations between e.s.d.'s in cell parameters are only used when they are defined by crystal symmetry. An approximate (isotropic) treatment of cell e.s.d.'s is used for estimating e.s.d.'s involving l.s. planes.
Refinement. Refinement of *F*^2^ against ALL reflections. The weighted *R*-factor *wR* and goodness of fit *S* are based on *F*^2^, conventional *R*-factors *R* are based on *F*, with *F* set to zero for negative *F*^2^. The threshold expression of *F*^2^ > σ(*F*^2^) is used only for calculating *R*-factors(gt) *etc*. and is not relevant to the choice of reflections for refinement. *R*-factors based on *F*^2^ are statistically about twice as large as those based on *F*, and *R*- factors based on ALL data will be even larger.

### Fractional atomic coordinates and isotropic or equivalent isotropic displacement parameters (Å^2^)

**Table:**

	*x*	*y*	*z*	*U*_iso_*/*U*_eq_	
Gd1	−0.046556 (10)	0.002766 (4)	0.340014 (8)	0.02550 (4)	
O1	0.03272 (16)	0.08532 (6)	0.30126 (14)	0.0388 (4)	
O2	0.12120 (18)	0.01332 (6)	0.28277 (15)	0.0436 (4)	
O3	0.0952 (2)	0.13429 (7)	0.15485 (15)	0.0528 (5)	
O4	0.08324 (17)	−0.07056 (6)	0.43696 (13)	0.0384 (4)	
O6	0.32842 (18)	−0.15230 (7)	0.65428 (16)	0.0521 (5)	
O7	−0.11813 (15)	−0.03158 (6)	0.48067 (12)	0.0337 (3)	
O8	−0.25649 (15)	−0.04212 (6)	0.30075 (12)	0.0346 (4)	
O9	−0.40902 (16)	−0.11065 (6)	0.34329 (14)	0.0432 (4)	
N1	−0.12084 (19)	−0.06791 (7)	0.18422 (15)	0.0326 (4)	
N2	−0.21912 (19)	0.03043 (7)	0.14094 (15)	0.0323 (4)	
C1	0.1032 (2)	0.06142 (9)	0.27068 (19)	0.0352 (5)	
C2	0.1743 (3)	0.09170 (10)	0.2203 (2)	0.0455 (6)	
H2A	0.1931	0.0685	0.1739	0.055*	
C3	0.3056 (3)	0.11432 (14)	0.3127 (3)	0.0679 (9)	
H3A	0.3490	0.1334	0.2803	0.102*	
H3B	0.3639	0.0868	0.3583	0.102*	
H3C	0.2871	0.1371	0.3580	0.102*	
C4	−0.0128 (3)	0.12487 (10)	0.0496 (2)	0.0440 (6)	
C5	−0.0846 (3)	0.16847 (12)	−0.0071 (3)	0.0577 (7)	
H5A	−0.0609	0.2007	0.0277	0.069*	
C6	−0.1906 (3)	0.16423 (14)	−0.1145 (3)	0.0700 (9)	
H6A	−0.2386	0.1937	−0.1525	0.084*	
C7	−0.2270 (3)	0.11702 (16)	−0.1669 (3)	0.0694 (9)	
H7A	−0.2990	0.1144	−0.2400	0.083*	
C8	−0.1560 (3)	0.07369 (14)	−0.1103 (3)	0.0655 (9)	
H8A	−0.1798	0.0417	−0.1458	0.079*	
C9	−0.0490 (3)	0.07709 (11)	−0.0010 (2)	0.0521 (7)	
H9A	−0.0025	0.0475	0.0376	0.063*	
C10	0.1572 (2)	−0.08655 (8)	0.53581 (18)	0.0307 (5)	
C11	0.2403 (2)	−0.13458 (9)	0.5442 (2)	0.0394 (6)	
H11A	0.1772	−0.1626	0.5023	0.047*	
C12	0.3250 (3)	−0.12420 (12)	0.4913 (3)	0.0610 (8)	
H12A	0.3728	−0.1551	0.4938	0.092*	
H12B	0.3887	−0.0971	0.5317	0.092*	
H12C	0.2674	−0.1138	0.4149	0.092*	
C13	0.2779 (3)	−0.18232 (9)	0.7067 (2)	0.0487 (7)	
C14	0.3765 (4)	−0.20978 (11)	0.7975 (3)	0.0701 (9)	
H14A	0.4662	−0.2085	0.8154	0.084*	
C15	0.3401 (6)	−0.23875 (15)	0.8606 (3)	0.0996 (15)	
H15A	0.4056	−0.2574	0.9215	0.120*	
C16	0.2094 (7)	−0.24059 (16)	0.8351 (4)	0.1070 (17)	
H16A	0.1859	−0.2602	0.8789	0.128*	
C17	0.1127 (5)	−0.21372 (13)	0.7455 (4)	0.0870 (12)	
H17A	0.0232	−0.2152	0.7282	0.104*	
C18	0.1463 (3)	−0.18461 (10)	0.6806 (3)	0.0580 (8)	
H18A	0.0799	−0.1665	0.6192	0.070*	
C19	−0.2279 (2)	−0.04794 (8)	0.39940 (17)	0.0269 (4)	
C20	−0.3273 (2)	−0.07336 (9)	0.42604 (19)	0.0359 (5)	
H20A	−0.2777	−0.0902	0.4994	0.043*	
C21	−0.4223 (3)	−0.03319 (11)	0.4272 (2)	0.0516 (7)	
H21A	−0.4863	−0.0497	0.4423	0.077*	
H21B	−0.4696	−0.0163	0.3557	0.077*	
H21C	−0.3715	−0.0080	0.4844	0.077*	
C22	−0.3458 (3)	−0.15389 (9)	0.3341 (2)	0.0463 (6)	
C23	−0.4223 (3)	−0.18490 (11)	0.2430 (3)	0.0632 (8)	
H23A	−0.5110	−0.1758	0.1913	0.076*	
C24	−0.3678 (4)	−0.22961 (13)	0.2280 (3)	0.0848 (11)	
H24A	−0.4201	−0.2503	0.1658	0.102*	
C25	−0.2378 (4)	−0.24390 (13)	0.3034 (4)	0.0945 (13)	
H25A	−0.2016	−0.2742	0.2933	0.113*	
C26	−0.1626 (4)	−0.21295 (14)	0.3934 (4)	0.1075 (16)	
H26A	−0.0742	−0.2224	0.4452	0.129*	
C27	−0.2152 (3)	−0.16744 (12)	0.4096 (3)	0.0825 (12)	
H27A	−0.1621	−0.1464	0.4710	0.099*	
C28	−0.2628 (2)	0.07881 (9)	0.1182 (2)	0.0390 (5)	
H28A	−0.2200	0.1036	0.1741	0.047*	
C29	−0.3701 (3)	0.09441 (10)	0.0145 (2)	0.0436 (6)	
H29A	−0.3968	0.1289	0.0018	0.052*	
C30	−0.4353 (3)	0.05859 (10)	−0.0678 (2)	0.0434 (6)	
H30A	−0.5084	0.0682	−0.1368	0.052*	
C31	−0.4547 (3)	−0.03305 (11)	−0.1292 (2)	0.0452 (6)	
H31A	−0.5297	−0.0252	−0.1985	0.054*	
C32	−0.4083 (3)	−0.08174 (11)	−0.1075 (2)	0.0472 (6)	
H32A	−0.4519	−0.1071	−0.1619	0.057*	
C33	−0.2382 (3)	−0.14554 (10)	0.0230 (2)	0.0505 (7)	
H33A	−0.2772	−0.1718	−0.0300	0.061*	
C34	−0.1286 (3)	−0.15558 (10)	0.1248 (2)	0.0530 (7)	
H34A	−0.0909	−0.1886	0.1420	0.064*	
C35	−0.0732 (3)	−0.11570 (9)	0.2033 (2)	0.0438 (6)	
H35A	0.0016	−0.1232	0.2729	0.053*	
C36	−0.3912 (2)	0.00715 (9)	−0.04773 (19)	0.0367 (5)	
C37	−0.2806 (2)	−0.00555 (8)	0.05864 (18)	0.0310 (5)	
C38	−0.2924 (2)	−0.09551 (9)	−0.0020 (2)	0.0385 (5)	
C39	−0.2296 (2)	−0.05740 (9)	0.08161 (18)	0.0314 (5)	
O5	0.16714 (16)	−0.06792 (6)	0.62245 (12)	0.0340 (3)	

### Atomic displacement parameters (Å^2^)

**Table:**

	*U*^11^	*U*^22^	*U*^33^	*U*^12^	*U*^13^	*U*^23^
Gd1	0.02465 (6)	0.02839 (6)	0.01925 (6)	0.00027 (4)	0.00808 (4)	0.00072 (4)
O1	0.0396 (10)	0.0372 (9)	0.0399 (9)	−0.0004 (7)	0.0203 (8)	0.0027 (7)
O2	0.0503 (11)	0.0383 (9)	0.0519 (11)	0.0013 (8)	0.0329 (9)	0.0048 (8)
O3	0.0616 (12)	0.0471 (10)	0.0390 (10)	−0.0111 (9)	0.0178 (10)	0.0090 (8)
O4	0.0462 (10)	0.0378 (9)	0.0242 (8)	0.0116 (7)	0.0127 (8)	0.0025 (7)
O6	0.0428 (11)	0.0496 (11)	0.0497 (11)	0.0096 (8)	0.0133 (9)	0.0110 (9)
O7	0.0293 (8)	0.0385 (8)	0.0237 (8)	−0.0044 (7)	0.0064 (7)	−0.0004 (6)
O8	0.0297 (8)	0.0464 (9)	0.0225 (8)	−0.0055 (7)	0.0094 (7)	−0.0015 (7)
O9	0.0298 (9)	0.0404 (9)	0.0476 (10)	−0.0058 (7)	0.0109 (8)	0.0004 (8)
N1	0.0314 (10)	0.0373 (10)	0.0262 (10)	0.0003 (8)	0.0125 (9)	−0.0023 (8)
N2	0.0329 (10)	0.0362 (10)	0.0239 (10)	0.0002 (8)	0.0117 (9)	0.0008 (8)
C1	0.0320 (12)	0.0433 (14)	0.0249 (12)	−0.0050 (10)	0.0105 (10)	0.0035 (10)
C2	0.0441 (15)	0.0527 (15)	0.0393 (14)	−0.0070 (12)	0.0210 (13)	0.0079 (12)
C3	0.0442 (17)	0.098 (2)	0.0511 (18)	−0.0250 (16)	0.0165 (15)	0.0163 (17)
C4	0.0463 (15)	0.0525 (15)	0.0346 (14)	−0.0082 (12)	0.0215 (13)	0.0074 (12)
C5	0.0595 (19)	0.0531 (17)	0.0593 (19)	−0.0025 (14)	0.0293 (16)	0.0123 (14)
C6	0.056 (2)	0.082 (2)	0.066 (2)	0.0077 (17)	0.0263 (18)	0.0301 (19)
C7	0.0472 (18)	0.116 (3)	0.0378 (17)	−0.0006 (19)	0.0163 (15)	0.0101 (18)
C8	0.060 (2)	0.085 (2)	0.0512 (19)	−0.0107 (17)	0.0287 (17)	−0.0189 (17)
C9	0.0541 (17)	0.0565 (17)	0.0445 (16)	0.0001 (13)	0.0241 (14)	0.0004 (13)
C10	0.0304 (12)	0.0302 (11)	0.0274 (12)	0.0010 (9)	0.0117 (10)	0.0019 (9)
C11	0.0399 (14)	0.0382 (13)	0.0333 (13)	0.0105 (10)	0.0136 (11)	0.0022 (10)
C12	0.0541 (18)	0.073 (2)	0.064 (2)	0.0201 (15)	0.0363 (17)	0.0061 (16)
C13	0.0655 (19)	0.0302 (12)	0.0480 (16)	0.0062 (12)	0.0270 (15)	0.0055 (11)
C14	0.076 (2)	0.0423 (16)	0.063 (2)	0.0036 (15)	0.0138 (18)	0.0039 (15)
C15	0.148 (4)	0.061 (2)	0.059 (2)	0.003 (3)	0.030 (3)	0.0189 (18)
C16	0.194 (6)	0.063 (2)	0.102 (4)	−0.002 (3)	0.102 (4)	0.014 (2)
C17	0.121 (3)	0.0499 (19)	0.124 (4)	−0.008 (2)	0.087 (3)	−0.002 (2)
C18	0.066 (2)	0.0373 (14)	0.070 (2)	0.0018 (13)	0.0340 (17)	0.0037 (13)
C19	0.0246 (11)	0.0255 (10)	0.0255 (12)	0.0014 (8)	0.0090 (10)	−0.0001 (8)
C20	0.0334 (13)	0.0408 (13)	0.0303 (12)	−0.0045 (10)	0.0139 (11)	0.0019 (10)
C21	0.0458 (16)	0.0627 (17)	0.0572 (18)	0.0027 (13)	0.0343 (15)	0.0000 (14)
C22	0.0404 (15)	0.0345 (13)	0.0549 (17)	−0.0067 (11)	0.0176 (13)	0.0042 (12)
C23	0.0571 (19)	0.0547 (18)	0.0582 (19)	−0.0047 (14)	0.0149 (16)	−0.0048 (15)
C24	0.097 (3)	0.055 (2)	0.081 (3)	−0.0070 (19)	0.029 (2)	−0.0203 (18)
C25	0.091 (3)	0.0490 (19)	0.121 (4)	0.0112 (19)	0.037 (3)	−0.014 (2)
C26	0.071 (3)	0.058 (2)	0.132 (4)	0.0205 (19)	0.007 (3)	−0.020 (2)
C27	0.052 (2)	0.0489 (18)	0.094 (3)	0.0084 (15)	−0.0011 (19)	−0.0149 (17)
C28	0.0427 (14)	0.0384 (13)	0.0303 (13)	0.0038 (11)	0.0145 (11)	0.0032 (10)
C29	0.0440 (15)	0.0464 (14)	0.0347 (14)	0.0108 (11)	0.0158 (12)	0.0116 (11)
C30	0.0358 (14)	0.0596 (16)	0.0251 (12)	0.0059 (12)	0.0085 (11)	0.0120 (11)
C31	0.0374 (14)	0.0665 (18)	0.0228 (12)	−0.0079 (12)	0.0090 (11)	−0.0009 (12)
C32	0.0434 (15)	0.0611 (17)	0.0294 (13)	−0.0159 (13)	0.0130 (12)	−0.0132 (12)
C33	0.0559 (17)	0.0470 (15)	0.0448 (16)	−0.0108 (13)	0.0229 (14)	−0.0176 (12)
C34	0.0628 (19)	0.0388 (14)	0.0511 (17)	0.0038 (13)	0.0244 (15)	−0.0076 (12)
C35	0.0456 (15)	0.0430 (14)	0.0350 (14)	0.0067 (11)	0.0147 (12)	−0.0012 (11)
C36	0.0321 (12)	0.0520 (14)	0.0239 (11)	−0.0031 (10)	0.0128 (10)	0.0025 (10)
C37	0.0279 (11)	0.0413 (12)	0.0234 (11)	−0.0033 (9)	0.0128 (9)	0.0010 (9)
C38	0.0392 (14)	0.0456 (14)	0.0322 (13)	−0.0089 (11)	0.0191 (11)	−0.0091 (11)
C39	0.0292 (12)	0.0416 (12)	0.0245 (11)	−0.0048 (9)	0.0143 (10)	−0.0032 (9)
O5	0.0390 (9)	0.0340 (8)	0.0255 (8)	0.0068 (7)	0.0139 (7)	0.0027 (7)

### Geometric parameters (Å, °)

**Table:**

Gd1—O7^i^	2.3528 (14)	C13—C18	1.365 (4)
Gd1—O4	2.3624 (15)	C13—C14	1.387 (4)
Gd1—O5^i^	2.3991 (15)	C14—C15	1.368 (6)
Gd1—O2	2.4443 (16)	C14—H14A	0.9300
Gd1—O8	2.4742 (15)	C15—C16	1.357 (6)
Gd1—O1	2.4826 (15)	C15—H15A	0.9300
Gd1—N2	2.5496 (18)	C16—C17	1.363 (6)
Gd1—N1	2.6184 (18)	C16—H16A	0.9300
Gd1—O7	2.6270 (15)	C17—C18	1.370 (4)
Gd1—Gd1^i^	4.0047 (2)	C17—H17A	0.9300
O1—C1	1.251 (3)	C18—H18A	0.9300
O2—C1	1.257 (3)	C19—C20	1.517 (3)
O3—C4	1.375 (3)	C20—C21	1.513 (3)
O3—C2	1.422 (3)	C20—H20A	0.9800
O4—C10	1.260 (3)	C21—H21A	0.9600
O6—C13	1.377 (3)	C21—H21B	0.9600
O6—C11	1.412 (3)	C21—H21C	0.9600
O7—C19	1.263 (2)	C22—C27	1.368 (4)
O7—Gd1^i^	2.3528 (14)	C22—C23	1.372 (4)
O8—C19	1.243 (2)	C23—C24	1.379 (5)
O9—C22	1.373 (3)	C23—H23A	0.9300
O9—C20	1.425 (3)	C24—C25	1.368 (5)
N1—C35	1.322 (3)	C24—H24A	0.9300
N1—C39	1.361 (3)	C25—C26	1.358 (5)
N2—C28	1.324 (3)	C25—H25A	0.9300
N2—C37	1.359 (3)	C26—C27	1.391 (5)
C1—C2	1.532 (3)	C26—H26A	0.9300
C2—C3	1.514 (4)	C27—H27A	0.9300
C2—H2A	0.9800	C28—C29	1.396 (3)
C3—H3A	0.9600	C28—H28A	0.9300
C3—H3B	0.9600	C29—C30	1.361 (4)
C3—H3C	0.9600	C29—H29A	0.9300
C4—C9	1.375 (4)	C30—C36	1.398 (3)
C4—C5	1.381 (4)	C30—H30A	0.9300
C5—C6	1.368 (4)	C31—C32	1.339 (4)
C5—H5A	0.9300	C31—C36	1.432 (3)
C6—C7	1.371 (5)	C31—H31A	0.9300
C6—H6A	0.9300	C32—C38	1.435 (4)
C7—C8	1.372 (5)	C32—H32A	0.9300
C7—H7A	0.9300	C33—C34	1.356 (4)
C8—C9	1.387 (4)	C33—C38	1.399 (4)
C8—H8A	0.9300	C33—H33A	0.9300
C9—H9A	0.9300	C34—C35	1.396 (4)
C10—O5	1.245 (3)	C34—H34A	0.9300
C10—C11	1.534 (3)	C35—H35A	0.9300
C11—C12	1.510 (4)	C36—C37	1.412 (3)
C11—H11A	0.9800	C37—C39	1.431 (3)
C12—H12A	0.9600	C38—C39	1.408 (3)
C12—H12B	0.9600	O5—Gd1^i^	2.3991 (15)
C12—H12C	0.9600		
			
O7^i^—Gd1—O4	73.51 (5)	C7—C8—C9	120.8 (3)
O7^i^—Gd1—O5^i^	77.96 (5)	C7—C8—H8A	119.6
O4—Gd1—O5^i^	134.90 (5)	C9—C8—H8A	119.6
O7^i^—Gd1—O2	88.00 (6)	C4—C9—C8	119.0 (3)
O4—Gd1—O2	84.30 (6)	C4—C9—H9A	120.5
O5^i^—Gd1—O2	129.04 (5)	C8—C9—H9A	120.5
O7^i^—Gd1—O8	123.43 (5)	O5—C10—O4	127.2 (2)
O4—Gd1—O8	90.69 (6)	O5—C10—C11	119.46 (19)
O5^i^—Gd1—O8	76.75 (5)	O4—C10—C11	113.38 (19)
O2—Gd1—O8	145.29 (5)	O6—C11—C12	107.3 (2)
O7^i^—Gd1—O1	76.76 (5)	O6—C11—C10	114.85 (19)
O4—Gd1—O1	128.11 (6)	C12—C11—C10	110.5 (2)
O5^i^—Gd1—O1	76.13 (5)	O6—C11—H11A	108.0
O2—Gd1—O1	52.93 (5)	C12—C11—H11A	108.0
O8—Gd1—O1	141.14 (5)	C10—C11—H11A	108.0
O7^i^—Gd1—N2	145.19 (6)	C11—C12—H12A	109.5
O4—Gd1—N2	139.42 (6)	C11—C12—H12B	109.5
O5^i^—Gd1—N2	79.73 (5)	H12A—C12—H12B	109.5
O2—Gd1—N2	85.65 (6)	C11—C12—H12C	109.5
O8—Gd1—N2	76.04 (5)	H12A—C12—H12C	109.5
O1—Gd1—N2	72.15 (6)	H12B—C12—H12C	109.5
O7^i^—Gd1—N1	147.42 (6)	C18—C13—O6	126.3 (2)
O4—Gd1—N1	75.96 (5)	C18—C13—C14	120.1 (3)
O5^i^—Gd1—N1	133.34 (5)	O6—C13—C14	113.5 (3)
O2—Gd1—N1	77.85 (6)	C15—C14—C13	119.1 (4)
O8—Gd1—N1	67.63 (5)	C15—C14—H14A	120.4
O1—Gd1—N1	114.94 (6)	C13—C14—H14A	120.4
N2—Gd1—N1	63.50 (6)	C16—C15—C14	120.6 (4)
O7^i^—Gd1—O7	73.06 (6)	C16—C15—H15A	119.7
O4—Gd1—O7	69.62 (5)	C14—C15—H15A	119.7
O5^i^—Gd1—O7	69.05 (5)	C15—C16—C17	120.0 (4)
O2—Gd1—O7	151.06 (5)	C15—C16—H16A	120.0
O8—Gd1—O7	50.74 (5)	C17—C16—H16A	120.0
O1—Gd1—O7	137.62 (5)	C16—C17—C18	120.5 (4)
N2—Gd1—O7	122.31 (5)	C16—C17—H17A	119.8
N1—Gd1—O7	106.49 (5)	C18—C17—H17A	119.8
O7^i^—Gd1—C1	83.56 (6)	C13—C18—C17	119.6 (3)
O4—Gd1—C1	107.99 (6)	C13—C18—H18A	120.2
O5^i^—Gd1—C1	102.59 (6)	C17—C18—H18A	120.2
O2—Gd1—C1	26.59 (6)	O8—C19—O7	121.83 (19)
O8—Gd1—C1	151.29 (6)	O8—C19—C20	120.67 (19)
O1—Gd1—C1	26.50 (6)	O7—C19—C20	117.46 (19)
N2—Gd1—C1	75.63 (6)	O8—C19—Gd1	57.39 (11)
N1—Gd1—C1	95.34 (6)	O7—C19—Gd1	64.45 (11)
O7—Gd1—C1	156.27 (6)	C20—C19—Gd1	177.62 (15)
O7^i^—Gd1—C19	98.61 (6)	O9—C20—C21	106.8 (2)
O4—Gd1—C19	79.26 (6)	O9—C20—C19	111.46 (18)
O5^i^—Gd1—C19	71.28 (5)	C21—C20—C19	110.04 (19)
O2—Gd1—C19	159.67 (6)	O9—C20—H20A	109.5
O8—Gd1—C19	25.04 (5)	C21—C20—H20A	109.5
O1—Gd1—C19	147.31 (5)	C19—C20—H20A	109.5
N2—Gd1—C19	99.11 (6)	C20—C21—H21A	109.5
N1—Gd1—C19	86.56 (6)	C20—C21—H21B	109.5
O7—Gd1—C19	25.70 (5)	H21A—C21—H21B	109.5
C1—Gd1—C19	172.75 (6)	C20—C21—H21C	109.5
O7^i^—Gd1—Gd1^i^	38.87 (4)	H21A—C21—H21C	109.5
O4—Gd1—Gd1^i^	66.72 (4)	H21B—C21—H21C	109.5
O5^i^—Gd1—Gd1^i^	69.07 (3)	C27—C22—C23	119.7 (3)
O2—Gd1—Gd1^i^	123.61 (4)	C27—C22—O9	124.2 (3)
O8—Gd1—Gd1^i^	84.75 (3)	C23—C22—O9	116.1 (2)
O1—Gd1—Gd1^i^	110.48 (4)	C22—C23—C24	120.0 (3)
N2—Gd1—Gd1^i^	146.41 (4)	C22—C23—H23A	120.0
N1—Gd1—Gd1^i^	132.94 (4)	C24—C23—H23A	120.0
O7—Gd1—Gd1^i^	34.20 (3)	C25—C24—C23	120.9 (3)
C1—Gd1—Gd1^i^	122.32 (5)	C25—C24—H24A	119.6
C19—Gd1—Gd1^i^	59.79 (4)	C23—C24—H24A	119.6
C1—O1—Gd1	91.23 (13)	C26—C25—C24	118.7 (3)
C1—O2—Gd1	92.88 (14)	C26—C25—H25A	120.6
C4—O3—C2	118.7 (2)	C24—C25—H25A	120.6
C10—O4—Gd1	139.77 (14)	C25—C26—C27	121.4 (4)
C13—O6—C11	119.4 (2)	C25—C26—H26A	119.3
C19—O7—Gd1^i^	162.02 (14)	C27—C26—H26A	119.3
C19—O7—Gd1	89.85 (12)	C22—C27—C26	119.3 (3)
Gd1^i^—O7—Gd1	106.94 (5)	C22—C27—H27A	120.3
C19—O8—Gd1	97.57 (12)	C26—C27—H27A	120.3
C22—O9—C20	117.49 (18)	N2—C28—C29	123.2 (2)
C35—N1—C39	117.5 (2)	N2—C28—H28A	118.4
C35—N1—Gd1	123.93 (16)	C29—C28—H28A	118.4
C39—N1—Gd1	117.70 (14)	C30—C29—C28	119.3 (2)
C28—N2—C37	118.3 (2)	C30—C29—H29A	120.3
C28—N2—Gd1	120.95 (15)	C28—C29—H29A	120.3
C37—N2—Gd1	120.28 (14)	C29—C30—C36	119.3 (2)
O1—C1—O2	122.2 (2)	C29—C30—H30A	120.3
O1—C1—C2	119.3 (2)	C36—C30—H30A	120.3
O2—C1—C2	118.4 (2)	C32—C31—C36	121.3 (2)
O1—C1—Gd1	62.28 (12)	C32—C31—H31A	119.4
O2—C1—Gd1	60.53 (12)	C36—C31—H31A	119.4
C2—C1—Gd1	174.03 (16)	C31—C32—C38	121.2 (2)
O3—C2—C3	106.3 (2)	C31—C32—H32A	119.4
O3—C2—C1	111.7 (2)	C38—C32—H32A	119.4
C3—C2—C1	110.1 (2)	C34—C33—C38	119.7 (2)
O3—C2—H2A	109.6	C34—C33—H33A	120.2
C3—C2—H2A	109.6	C38—C33—H33A	120.2
C1—C2—H2A	109.6	C33—C34—C35	119.0 (2)
C2—C3—H3A	109.5	C33—C34—H34A	120.5
C2—C3—H3B	109.5	C35—C34—H34A	120.5
H3A—C3—H3B	109.5	N1—C35—C34	123.7 (2)
C2—C3—H3C	109.5	N1—C35—H35A	118.1
H3A—C3—H3C	109.5	C34—C35—H35A	118.1
H3B—C3—H3C	109.5	C30—C36—C37	118.1 (2)
O3—C4—C9	125.2 (2)	C30—C36—C31	123.0 (2)
O3—C4—C5	114.6 (2)	C37—C36—C31	118.9 (2)
C9—C4—C5	120.2 (3)	N2—C37—C36	121.8 (2)
C6—C5—C4	119.9 (3)	N2—C37—C39	118.29 (19)
C6—C5—H5A	120.1	C36—C37—C39	119.9 (2)
C4—C5—H5A	120.1	C33—C38—C39	117.7 (2)
C5—C6—C7	120.7 (3)	C33—C38—C32	123.1 (2)
C5—C6—H6A	119.6	C39—C38—C32	119.2 (2)
C7—C6—H6A	119.6	N1—C39—C38	122.4 (2)
C6—C7—C8	119.4 (3)	N1—C39—C37	118.26 (19)
C6—C7—H7A	120.3	C38—C39—C37	119.4 (2)
C8—C7—H7A	120.3	C10—O5—Gd1^i^	134.51 (14)
			
O7^i^—Gd1—O1—C1	102.35 (14)	O8—Gd1—C1—O2	−100.11 (17)
O4—Gd1—O1—C1	46.07 (15)	O1—Gd1—C1—O2	171.6 (2)
O5^i^—Gd1—O1—C1	−177.01 (14)	N2—Gd1—C1—O2	−109.69 (14)
O2—Gd1—O1—C1	4.72 (13)	N1—Gd1—C1—O2	−48.80 (14)
O8—Gd1—O1—C1	−130.06 (13)	O7—Gd1—C1—O2	108.27 (18)
N2—Gd1—O1—C1	−93.53 (14)	Gd1^i^—Gd1—C1—O2	101.51 (14)
N1—Gd1—O1—C1	−45.34 (14)	C4—O3—C2—C3	163.1 (2)
O7—Gd1—O1—C1	147.77 (12)	C4—O3—C2—C1	−76.8 (3)
C19—Gd1—O1—C1	−172.36 (13)	O1—C1—C2—O3	−35.4 (3)
Gd1^i^—Gd1—O1—C1	122.00 (12)	O2—C1—C2—O3	146.6 (2)
O7^i^—Gd1—O2—C1	−79.58 (14)	O1—C1—C2—C3	82.4 (3)
O4—Gd1—O2—C1	−153.20 (14)	O2—C1—C2—C3	−95.5 (3)
O5^i^—Gd1—O2—C1	−6.86 (17)	C2—O3—C4—C9	−2.7 (4)
O8—Gd1—O2—C1	123.85 (14)	C2—O3—C4—C5	179.2 (2)
O1—Gd1—O2—C1	−4.70 (13)	O3—C4—C5—C6	177.1 (3)
N2—Gd1—O2—C1	66.17 (14)	C9—C4—C5—C6	−1.1 (4)
N1—Gd1—O2—C1	129.97 (15)	C4—C5—C6—C7	0.2 (5)
O7—Gd1—O2—C1	−127.86 (14)	C5—C6—C7—C8	0.1 (5)
C19—Gd1—O2—C1	170.75 (16)	C6—C7—C8—C9	0.5 (5)
Gd1^i^—Gd1—O2—C1	−96.11 (14)	O3—C4—C9—C8	−176.3 (3)
O7^i^—Gd1—O4—C10	24.2 (2)	C5—C4—C9—C8	1.8 (4)
O5^i^—Gd1—O4—C10	−28.8 (3)	C7—C8—C9—C4	−1.5 (5)
O2—Gd1—O4—C10	113.8 (2)	Gd1—O4—C10—O5	13.8 (4)
O8—Gd1—O4—C10	−100.7 (2)	Gd1—O4—C10—C11	−166.83 (17)
O1—Gd1—O4—C10	81.8 (2)	C13—O6—C11—C12	−155.8 (2)
N2—Gd1—O4—C10	−169.8 (2)	C13—O6—C11—C10	81.0 (3)
N1—Gd1—O4—C10	−167.4 (2)	O5—C10—C11—O6	−3.0 (3)
O7—Gd1—O4—C10	−53.5 (2)	O4—C10—C11—O6	177.6 (2)
C1—Gd1—O4—C10	101.5 (2)	O5—C10—C11—C12	−124.5 (2)
C19—Gd1—O4—C10	−78.3 (2)	O4—C10—C11—C12	56.0 (3)
Gd1^i^—Gd1—O4—C10	−16.7 (2)	C11—O6—C13—C18	−22.0 (4)
O7^i^—Gd1—O7—C19	173.47 (15)	C11—O6—C13—C14	161.6 (2)
O4—Gd1—O7—C19	−108.27 (13)	C18—C13—C14—C15	−0.4 (5)
O5^i^—Gd1—O7—C19	90.18 (12)	O6—C13—C14—C15	176.3 (3)
O2—Gd1—O7—C19	−135.29 (13)	C13—C14—C15—C16	−0.2 (6)
O8—Gd1—O7—C19	0.41 (11)	C14—C15—C16—C17	0.5 (7)
O1—Gd1—O7—C19	127.01 (12)	C15—C16—C17—C18	−0.2 (6)
N2—Gd1—O7—C19	28.09 (14)	O6—C13—C18—C17	−175.5 (3)
N1—Gd1—O7—C19	−40.59 (13)	C14—C13—C18—C17	0.7 (4)
C1—Gd1—O7—C19	163.26 (16)	C16—C17—C18—C13	−0.4 (5)
Gd1^i^—Gd1—O7—C19	173.47 (15)	Gd1—O8—C19—O7	0.8 (2)
O7^i^—Gd1—O7—Gd1^i^	0.0	Gd1—O8—C19—C20	178.39 (17)
O4—Gd1—O7—Gd1^i^	78.27 (6)	Gd1^i^—O7—C19—O8	158.6 (3)
O5^i^—Gd1—O7—Gd1^i^	−83.28 (6)	Gd1—O7—C19—O8	−0.8 (2)
O2—Gd1—O7—Gd1^i^	51.24 (13)	Gd1^i^—O7—C19—C20	−19.1 (6)
O8—Gd1—O7—Gd1^i^	−173.05 (9)	Gd1—O7—C19—C20	−178.41 (17)
O1—Gd1—O7—Gd1^i^	−46.45 (10)	Gd1^i^—O7—C19—Gd1	159.3 (5)
N2—Gd1—O7—Gd1^i^	−145.37 (6)	O7^i^—Gd1—C19—O8	172.92 (12)
N1—Gd1—O7—Gd1^i^	145.94 (6)	O4—Gd1—C19—O8	−115.80 (13)
C1—Gd1—O7—Gd1^i^	−10.20 (18)	O5^i^—Gd1—C19—O8	98.83 (13)
C19—Gd1—O7—Gd1^i^	−173.47 (15)	O2—Gd1—C19—O8	−79.2 (2)
O7^i^—Gd1—O8—C19	−8.40 (15)	O1—Gd1—C19—O8	94.06 (15)
O4—Gd1—O8—C19	62.20 (13)	N2—Gd1—C19—O8	23.01 (13)
O5^i^—Gd1—O8—C19	−74.05 (12)	N1—Gd1—C19—O8	−39.45 (13)
O2—Gd1—O8—C19	143.17 (13)	O7—Gd1—C19—O8	179.2 (2)
O1—Gd1—O8—C19	−120.83 (13)	Gd1^i^—Gd1—C19—O8	175.00 (14)
N2—Gd1—O8—C19	−156.56 (13)	O7^i^—Gd1—C19—O7	−6.32 (15)
N1—Gd1—O8—C19	136.70 (14)	O4—Gd1—C19—O7	64.96 (12)
O7—Gd1—O8—C19	−0.42 (11)	O5^i^—Gd1—C19—O7	−80.41 (12)
C1—Gd1—O8—C19	−166.13 (14)	O2—Gd1—C19—O7	101.55 (19)
Gd1^i^—Gd1—O8—C19	−4.34 (12)	O8—Gd1—C19—O7	−179.2 (2)
O7^i^—Gd1—N1—C35	21.8 (2)	O1—Gd1—C19—O7	−85.18 (15)
O4—Gd1—N1—C35	0.91 (19)	N2—Gd1—C19—O7	−156.23 (12)
O5^i^—Gd1—N1—C35	−138.95 (18)	N1—Gd1—C19—O7	141.31 (12)
O2—Gd1—N1—C35	88.00 (19)	Gd1^i^—Gd1—C19—O7	−4.24 (10)
O8—Gd1—N1—C35	−95.8 (2)	C22—O9—C20—C21	−174.0 (2)
O1—Gd1—N1—C35	126.74 (19)	C22—O9—C20—C19	65.8 (3)
N2—Gd1—N1—C35	179.2 (2)	O8—C19—C20—O9	30.7 (3)
O7—Gd1—N1—C35	−62.4 (2)	O7—C19—C20—O9	−151.64 (19)
C1—Gd1—N1—C35	108.15 (19)	O8—C19—C20—C21	−87.6 (3)
C19—Gd1—N1—C35	−78.86 (19)	O7—C19—C20—C21	90.1 (2)
Gd1^i^—Gd1—N1—C35	−37.0 (2)	C20—O9—C22—C27	10.8 (4)
O7^i^—Gd1—N1—C39	−169.06 (13)	C20—O9—C22—C23	−170.1 (2)
O4—Gd1—N1—C39	170.09 (16)	C27—C22—C23—C24	0.3 (5)
O5^i^—Gd1—N1—C39	30.23 (18)	O9—C22—C23—C24	−178.9 (3)
O2—Gd1—N1—C39	−102.83 (16)	C22—C23—C24—C25	0.4 (6)
O8—Gd1—N1—C39	73.41 (15)	C23—C24—C25—C26	−0.5 (7)
O1—Gd1—N1—C39	−64.09 (16)	C24—C25—C26—C27	−0.1 (8)
N2—Gd1—N1—C39	−11.64 (14)	C23—C22—C27—C26	−0.8 (6)
O7—Gd1—N1—C39	106.74 (15)	O9—C22—C27—C26	178.2 (4)
C1—Gd1—N1—C39	−82.67 (16)	C25—C26—C27—C22	0.8 (7)
C19—Gd1—N1—C39	90.32 (15)	C37—N2—C28—C29	1.0 (4)
Gd1^i^—Gd1—N1—C39	132.20 (13)	Gd1—N2—C28—C29	−170.91 (18)
O7^i^—Gd1—N2—C28	−18.1 (2)	N2—C28—C29—C30	0.9 (4)
O4—Gd1—N2—C28	−174.25 (16)	C28—C29—C30—C36	−1.6 (4)
O5^i^—Gd1—N2—C28	32.72 (17)	C36—C31—C32—C38	−0.4 (4)
O2—Gd1—N2—C28	−98.25 (18)	C38—C33—C34—C35	0.9 (4)
O8—Gd1—N2—C28	111.48 (18)	C39—N1—C35—C34	−0.5 (4)
O1—Gd1—N2—C28	−45.88 (17)	Gd1—N1—C35—C34	168.7 (2)
N1—Gd1—N2—C28	−176.84 (19)	C33—C34—C35—N1	−0.5 (4)
O7—Gd1—N2—C28	89.72 (18)	C29—C30—C36—C37	0.6 (4)
C1—Gd1—N2—C28	−73.25 (18)	C29—C30—C36—C31	179.4 (2)
C19—Gd1—N2—C28	101.66 (18)	C32—C31—C36—C30	178.9 (2)
Gd1^i^—Gd1—N2—C28	54.5 (2)	C32—C31—C36—C37	−2.3 (4)
O7^i^—Gd1—N2—C37	170.22 (14)	C28—N2—C37—C36	−2.0 (3)
O4—Gd1—N2—C37	14.0 (2)	Gd1—N2—C37—C36	169.90 (16)
O5^i^—Gd1—N2—C37	−138.98 (16)	C28—N2—C37—C39	177.3 (2)
O2—Gd1—N2—C37	90.05 (16)	Gd1—N2—C37—C39	−10.7 (3)
O8—Gd1—N2—C37	−60.22 (16)	C30—C36—C37—N2	1.3 (3)
O1—Gd1—N2—C37	142.42 (17)	C31—C36—C37—N2	−177.6 (2)
N1—Gd1—N2—C37	11.46 (15)	C30—C36—C37—C39	−178.1 (2)
O7—Gd1—N2—C37	−81.98 (16)	C31—C36—C37—C39	3.1 (3)
C1—Gd1—N2—C37	115.05 (17)	C34—C33—C38—C39	−0.3 (4)
C19—Gd1—N2—C37	−70.04 (16)	C34—C33—C38—C32	−179.9 (3)
Gd1^i^—Gd1—N2—C37	−117.23 (15)	C31—C32—C38—C33	−178.1 (3)
Gd1—O1—C1—O2	−8.7 (2)	C31—C32—C38—C39	2.3 (4)
Gd1—O1—C1—C2	173.45 (19)	C35—N1—C39—C38	1.1 (3)
Gd1—O2—C1—O1	8.8 (2)	Gd1—N1—C39—C38	−168.80 (16)
Gd1—O2—C1—C2	−173.29 (18)	C35—N1—C39—C37	−178.6 (2)
O7^i^—Gd1—C1—O1	−73.12 (13)	Gd1—N1—C39—C37	11.5 (2)
O4—Gd1—C1—O1	−143.43 (12)	C33—C38—C39—N1	−0.7 (3)
O5^i^—Gd1—C1—O1	2.97 (14)	C32—C38—C39—N1	178.9 (2)
O2—Gd1—C1—O1	−171.6 (2)	C33—C38—C39—C37	178.9 (2)
O8—Gd1—C1—O1	88.32 (17)	C32—C38—C39—C37	−1.5 (3)
N2—Gd1—C1—O1	78.74 (13)	N2—C37—C39—N1	−0.9 (3)
N1—Gd1—C1—O1	139.62 (13)	C36—C37—C39—N1	178.46 (19)
O7—Gd1—C1—O1	−63.3 (2)	N2—C37—C39—C38	179.4 (2)
Gd1^i^—Gd1—C1—O1	−70.07 (14)	C36—C37—C39—C38	−1.2 (3)
O7^i^—Gd1—C1—O2	98.45 (14)	O4—C10—O5—Gd1^i^	8.9 (4)
O4—Gd1—C1—O2	28.14 (15)	C11—C10—O5—Gd1^i^	−170.47 (15)
O5^i^—Gd1—C1—O2	174.55 (13)		

Symmetry codes: (i) −*x*, −*y*, −*z*+1.
